# Supplementary material for: A novel truncating variant of GLI2 associated with Culler-Jones syndrome impairs Hedgehog signalling
Source: PLoS One. 2019 Jan 10;14(1):e0210097. doi: 10.1371/journal.pone.0210097 (PMC6328167; doi:10.1371/journal.pone.0210097)
Supplement: S1 Table — Main sequencing statistics for each sample. All samples were sequenced at 30x coverage minimum. (DOCX) [file pone.0210097.s001.docx]

| Sample | Total reads | % duplicate | On Target Coverage |
| --- | --- | --- | --- |
| 01 | 26569539 | 8.9% | 31x |
| 02 | 29422240 | 9.3% | 35x |
| 03 | 26214054 | 9.4% | 31x |
