# Supplementary material for: A novel truncating variant of GLI2 associated with Culler-Jones syndrome impairs Hedgehog signalling
Source: PLoS One. 2019 Jan 10;14(1):e0210097. doi: 10.1371/journal.pone.0210097 (PMC6328167; doi:10.1371/journal.pone.0210097)
Supplement: S2 Table — Genomic positions and annotation of variants selected for disease gene prioritization. Only rare, putative damaging variants segregating in the pedigree were selected. Annotation was performed on GRCh37.34 genome version. (DOCX) [file pone.0210097.s002.docx]

| Chr | Position | Reference  Allele | Alternate  Allele | Gene  Symbol | Effect | Nucleotide  Change | Aminoacid Change |
| --- | --- | --- | --- | --- | --- | --- | --- |
| chr1 | 3782550 | C | T | DFFB | NON_SYNONYMOUS_CODING | cCg/cTg | P139L |
| chr1 | 5924056 | C | T | NPHP4 | NON_SYNONYMOUS_CODING | gGt/gAt | G1345D |
| chr1 | 186024710 | C | A | HMCN1 | NON_SYNONYMOUS_CODING | Ctg/Atg | L2350M |
| chr1 | 205683591 | TAC | T | AC119673.1 | FRAME_SHIFT | -/- | -41 |
| chr1 | 246797823 | A | G | CNST | NON_SYNONYMOUS_CODING | aAt/aGt | N251S |
| chr10 | 35928500 | A | G | FZD8 | NON_SYNONYMOUS_CODING | Tgc/Cgc | C620R |
| chr10 | 46246238 | G | A | FAM21C | NON_SYNONYMOUS_CODING | Gcc/Acc | A295T |
| chr11 | 76954788 | T | TA | GDPD4 | FRAME_SHIFT | -/T | -397? |
| chr11 | 108141988 | T | C | ATM | NON_SYNONYMOUS_CODING | Tct/Cct | S978P |
| chr11 | 118967712 | A | C | DPAGT1 | NON_SYNONYMOUS_CODING | gTc/gGc | V408G |
| chr12 | 14947563 | T | C | WBP11 | NON_SYNONYMOUS_CODING | cAa/cGa | Q210R |
| chr12 | 46318555 | G | T | SCAF11 | NON_SYNONYMOUS_CODING | Caa/Aaa | Q1288K |
| chr12 | 48189531 | G | T | HDAC7 | NON_SYNONYMOUS_CODING | aCc/aAc | T326N |
| chr12 | 55714406 | C | CA | OR6C1 | FRAME_SHIFT | -/A | -8? |
| chr12 | 108912521 | C | T | FICD | NON_SYNONYMOUS_CODING | Cgc/Tgc | R216C |
| chr14 | 75514662 | G | T | MLH3 | NON_SYNONYMOUS_CODING | cCt/cAt | P566H |
| chr15 | 33999253 | A | C | RYR3 | NON_SYNONYMOUS_CODING | aAc/aCc | N2206T |
| chr17 | 34871001 | T | TG | MYO19 | FRAME_SHIFT | -/C | -191? |
| chr17 | 45438886 | CAGTG | C | EFCAB13 | FRAME_SHIFT | -/- | -269 |
| chr19 | 9236698 | G | GATGGT | OR7G3 | FRAME_SHIFT | -/ACCAT | -310T? |
| chr19 | 50398373 | G | A | IL4I1 | NON_SYNONYMOUS_CODING | aCg/aTg | T128M |
| chr19 | 55494955 | T | C | NLRP2 | NON_SYNONYMOUS_CODING | cTg/cCg | L630P |
| chr2 | 121746982 | AC | A | GLI2 | FRAME_SHIFT | -/- | -1165 |
| chr2 | 180383287 | A | G | ZNF385B | NON_SYNONYMOUS_CODING | Tgt/Cgt | C159R |
| chr20 | 31017824 | C | T | ASXL1 | NON_SYNONYMOUS_CODING | cCg/cTg | P229L |
| chr22 | 37904616 | G | A | CARD10 | NON_SYNONYMOUS_CODING | gCg/gTg | A328V |
| chr3 | 17413575 | T | C | TBC1D5 | NON_SYNONYMOUS_CODING | atA/atG | I329M |
| chr3 | 56591278 | T | TGGGGTAAGCA | CCDC66 | FRAME_SHIFT | -/GGGGTAAGCA | -3GVS? |
| chr3 | 75790814 | C | CT | ZNF717 | FRAME_SHIFT | -/A | -44? |
| chr3 | 100527069 | C | G | ABI3BP | NON_SYNONYMOUS_CODING | caG/caC | Q1213H |
| chr3 | 108475993 | G | GGGGGATTA | RETNLB | FRAME_SHIFT | -/TAATCCCC | -13*S? |
| chr6 | 24476437 | C | T | GPLD1 | NON_SYNONYMOUS_CODING | cGa/cAa | R101Q |
| chr6 | 137245785 | A | C | SLC35D3 | NON_SYNONYMOUS_CODING | tAt/tCt | Y401S |
| chr7 | 12391268 | T | TA | VWDE | FRAME_SHIFT | -/T | -1272? |
| chr7 | 82585368 | T | A | PCLO | NON_SYNONYMOUS_CODING | gAt/gTt | D1634V |
| chr7 | 116918397 | G | A | WNT2 | NON_SYNONYMOUS_CODING | Cgg/Tgg | R299W |
| chr7 | 143771322 | A | T | OR2A25 | NON_SYNONYMOUS_CODING | Aat/Tat | N4Y |
| chr8 | 20005123 | A | G | SLC18A1 | NON_SYNONYMOUS_CODING | Ttt/Ctt | F441L |
| chr9 | 125391770 | C | CA | OR1B1 | FRAME_SHIFT | -/T | -15? |
| chr9 | 131418941 | G | A | WDR34 | NON_SYNONYMOUS_CODING | gCg/gTg | A22V |
